# Genetics, cardiac phenotype and cardiovascular outcomes in Fabry disease patients in Finland

**DOI:** 10.1002/ehf2.15387

**Published:** 2025-07-21

**Authors:** Kati Valtola, Päivi Pietilä‐Effati, Jonna M. E. Männistö, Susanne Walls, Ilkka Kantola, Johanna Kuusisto

**Affiliations:** ^1^ Heart Center of Kuopio University Hospital Kuopio Finland; ^2^ Vaasa Central Hospital Vaasa Finland; ^3^ Department of Clinical Genetics Kuopio University Hospital Kuopio Finland; ^4^ Division of Medicine Turku University Hospital, Turku University Turku Finland; ^5^ School of Medicine University of Turku Turku Finland; ^6^ Centre for Medicine and Clinical Research University of Eastern Finland and Kuopio University Hospital Kuopio Finland

**Keywords:** Cardiac imaging, Cardiomyopathy, Fabry disease, GLA, Outcomes

## Abstract

**Aims:**

To investigate the genetics, cardiac phenotype and cardiovascular outcomes of Finnish Fabry patients.

**Methods and results:**

Among the 109 patients with Fabry disease (FD) diagnosed in Finland by 2018, 97 (89%; 32 males and 65 females, mean ages 42 and 52 years) were followed for a mean of 12 years. Data on genetics, phenotypes, cardiac imaging and cardiovascular outcomes were collected from the Fabry Registry and medical records. The 26 families with FD harboured 22 different hemi−/heterozygous *GLA* variants, most commonly p.R227X, p.A143T or p.P409A. The Fabry phenotype in males was classic in 19 (59%), late‐onset in 10 (31%) and intermediate in 3 (9%) patients. Among the females, 62 (95%) were symptomatic. Fabry cardiomyopathy (FC, maximal left ventricular wall thickness ≥13 mm, or an increased cardiac mass and decreased T1 time, or typical late gadolinium enhancement (LGE) in CMR) was present in 21 (66%) males manifesting since their 20s, and in 32 (49%) females since their 40s. LGE in CMR was detected in most subjects with cardiomyopathy, particularly in females. Among the 53 patients with FC, 16 (30%) developed atrial fibrillation, 17 (32%) stroke, 14 (26%) heart failure (HF) and 3 (6%) end‐stage renal disease. Nine patients died during the follow‐up at mean ages of 48 (males) and 75 years (females), three of whom died from HF and three from stroke. Eight of those who died had cardiomyopathy.

**Conclusions:**

In Finland, FD is caused by multiple *GLA* variants. Classic phenotype is more common. Contrasting previous studies, most women are symptomatic. Cardiomyopathy is very common also in women since their 40s and associates with atrial fibrillation, HF, stroke and death, emphasizing the malignant natural course of FC. Our findings highlight the need for even more diligent monitoring of cardiac manifestations also in females with FD by regular cardiac imaging with CMR.

## Introduction

Fabry disease (FD) OMIM: #301500) is a rare and underdiagnosed lysosomal storage disease with an estimated prevalence of 0.01% in the general population.^1^ FD is caused by a low or absent level of α‐galactosidase A enzyme (α‐Gal A) due to genetic defects in the α‐galactosidase A gene (*GLA*) on the X chromosome (Xq22.1). Enzyme deficiency leads to the accumulation of sphingolipids, mainly globotriaosylceramide (Gb_3_) and lysoglobotriaosylceramide (lyso‐Gb_3_), in the lysosomes of cells in multiple organs. FD is a progressive disease that, if untreated, significantly reduces quality of life and leads to premature death. FD can be treated with specific enzyme replacement therapy (ERT), or in some cases, chaperone therapy migalastat. However, for the treatment to be effective, it should be started before sphingolipid accumulation has caused extensive fibrosis in the organs.^2^, ^3^

More than 1000 different variants in the *GLA* gene have been reported. Most of them are identified in a single family. In addition phenotype of FD is often nonspesific, which makes interpretation of the pathogenicity and phenotype of genetic variants challenging.^1^, ^2^, ^4^ Due to X‐linked dominant inheritance pattern, hemizygous males always develop some degree of FD, whereas the phenotype of heterozygous females can range from as severe as that of males to asymptomatic.^5^, ^6^

The classic form of FD is caused by pathogenic *GLA* variants that induce the absence or near absence α‐Gal A activity in hemizygous males. Classic FD patients suffer from severe intermittent limb pain, a lack of sweating, gastrointestinal problems, and fatigue beginning in early childhood. Angiokeratomas and cornea verticillata may be present during childhood. If left untreated, affected males in their 30s and females in their 40s usually develop multiorgan disease, including kidney failure, stroke and Fabry cardiomyopathy (FC). The more common late‐onset form of FD is caused by *GLA* variants which allow for over 5% residual α‐Gal A activity in males. Both sexes can be affected, and often, the most prominent and only manifestation is cardiomyopathy in the middle age.^6^, ^7^, ^8^ In addition to the effects of the *GLA* variant, sex, age and yet unknown factors contribute to the phenotype of FD, which can vary even between males in the same family.^9^ The plasma levels of α‐Gal A and lyso‐Gb_3_ predict, at least to some extent, the severity of the disease in a *GLA* variant carrier.^10^

To our knowledge, there is only one previous comprehensive nationwide study from Iceland on the genotype and cardiac phenotype of FD.^11^ The Finnish population differs from others in terms of its unique genetic architecture and its own set of hereditary diseases.^12^ Nearly all Finnish patients diagnosed with FD have been included in the national Fabry Registry, which is filled comprehensively by a nationwide network of Fabry experts, who actively follow the national and international guidelines of FD. Therefore, we conducted the present study to investigate the genetics, phenotypes, cardiac imaging findings and cardiovascular outcomes of Finnish Fabry Registry patients. As cardiac causes are the leading cause of death in Fabry patients,^10^, ^13^ our focus was particularly on FC and its natural course. Finally, as FC is the most common manifestation of late‐onset FD, the aim of this study was to improve cardiologists' knowledge of FC.

## Methods

### Setting

The Fabry Disease Centre of Excellence was established at Turku University Hospital in 2002. The Centre maintains the Fabry Registry in Finland. The Fabry Registry (ClinicalTrials.gov Identifier NCT00196742) is a multicentre, international, longitudinal, patient‐centred, observational study open to all patients with a confirmed diagnosis of FD.^14^

**Figure 1:**
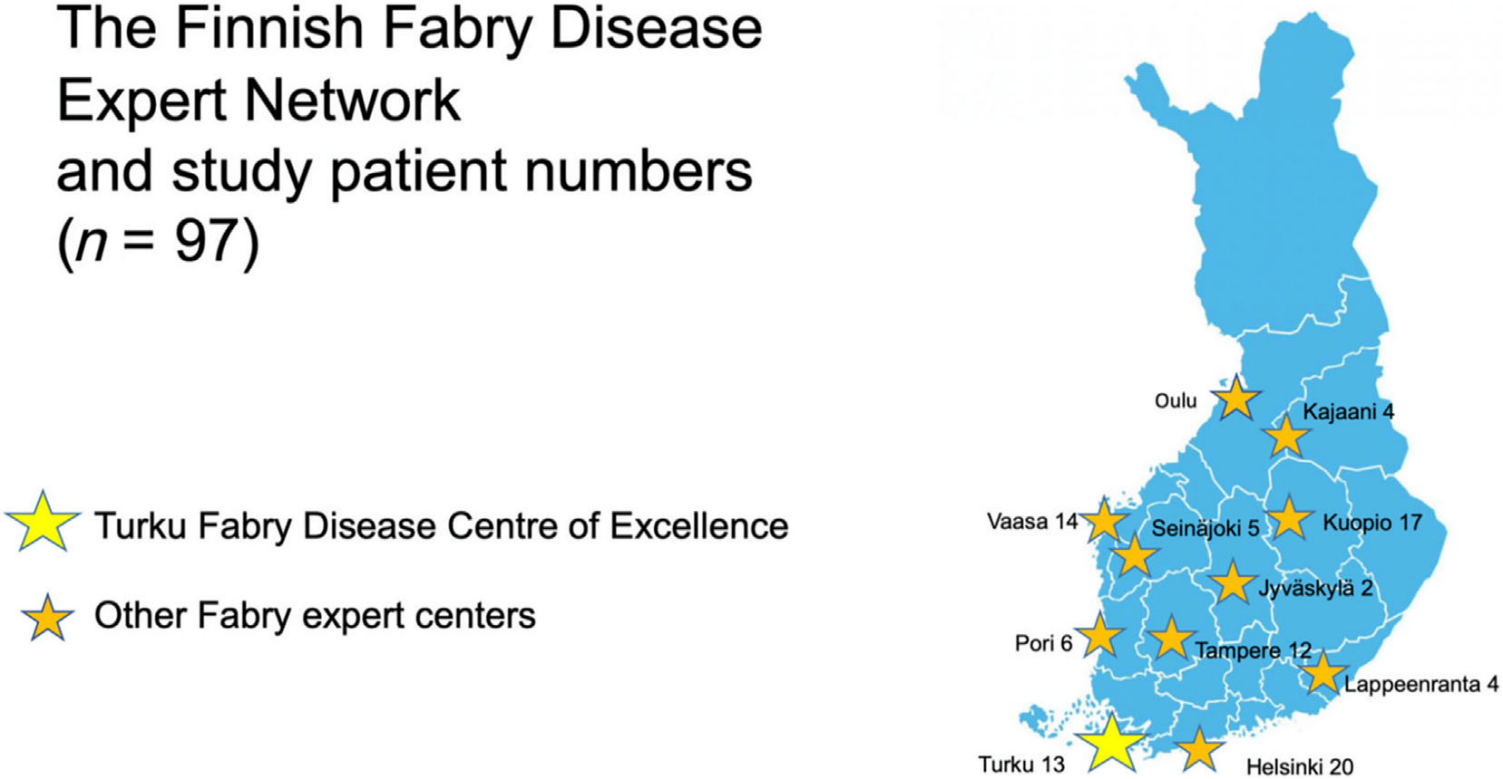
The Finnish Fabry disease expert network and study patient numbers.

Currently, the treatment and follow‐up of Fabry patients in Finland are centralized in five university hospitals and a few central hospitals, with FD expertise. Among all 109 Finnish patients diagnosed with FD and/or pathogenic or likely pathogenic variants in *GLA* from 1972 to 2018, a total of 97 (89%) patients from 26 families were included in the Fabry Registry and the present study (see *Figure* *1* and Data S1). When the index patient was diagnosed with FD, systematic cascade screening of the disease‐causing variant was carried out in family members. Many late‐onset FD cases were found when patients with hypertrophic cardiomyopathy were investigated by cardiac magnetic resonance (CMR) and genetic testing by comprehensive cardiomyopathy gene panels, including the GLA gene, as suggested by the current 2023 ESC cardiomyopathy guidelines.

### Data sources

The data for this study were collected from both the Fabry Registry and the patients' hospital medical records by cardiologists with FD expertise until 17 October 2018. In addition, major cardiovascular and renal events, and cardiac imaging data were recorded from the Fabry Registry until the end of 2019.

### Genetic data

The genetic analyses of the *GLA* were performed in a clinical setting either in the Finnish Functional Genomics Centre in Turku, Genome Center of Eastern Finland in Kuopio, Blueprint Genetics Laboratory in Espoo, Finland, ARCHIMEDlife Laboratories in Vienna, Austria, or the laboratory of Academic Medical Centre of University of Amsterdam, the Netherlands. The genetic data were available for all the study subjects. In this study, the pathogenicity of the variants was reassessed. Variants were annotated by AlaMut batch software V.1.8 (Interactive Biosoftware, France) and by the transcript NM 000169.2 Pathogenicity was assessed according to guidelines for the interpretation of single nucleotide variants by the American College of Medical Genetics (ACMG) and guidelines for the interpretation of copy number variants by the ACMG and Clinical Genome Resources (ClinGen).^15^, ^16^

### Fabry disease phenotype classification

In this study, the FD phenotype in male patients was classified as classic, intermediate or late‐onset by cardiologists and internists participating in the present study on the basis of characteristic symptoms, age at onset, long‐term disease manifestations, enzyme activity and the type of disease‐causing genetic variant as suggested by European FD experts^7^, ^17^, ^18^, ^19^ (see Data S1). The phenotype was not definable if only females were affected. Females were classified as either symptomatic or asymptomatic.

### Alpha‐galactosidase A enzyme activity and lyso‐Gb_3_ level

See Data S1.

### Cardiac imaging

See Data S1.

### FC diagnosis

According to current ESC Guidelines for the management of cardiomyopathies, the diagnostic criterion for FC is maximal left ventricular wall thickness of 13 mm or greater on cardiac imaging measured at the end‐diastole in a patient with FD and no other reason for left ventricular hypertrophy, which we used as the FC criterion in our study.^18^ In a family with the *GLA* p.A143T variant and cardiomyopathy, FC was confirmed by histological and immunohistochemical analyses of endomyocardial biopsies.^20^ In addition, for one female with *GLA* p.T410A, and another with p.R227X variant, an increased cardiac mass and decreased T1 time or typical LGE on CMR, respectively, were considered sufficient criteria for FC, as suggested by recent literature, although LV maximal wall thickness was under 13 mm.^9^, ^13^

### Cardiovascular outcomes and mortality

Cardiovascular events and deaths of patients were carefully reviewed on a case‐by‐case basis from medical records, laboratory test results, ECGs, X‐ray reports and pathology reports by cardiologists with FD expertise.

### Statistical analyses

Continuous variables are presented as the means with standard deviations. Categorical variables are shown as frequencies with percentages. Continuous variables were statistically compared by independent samples t‐tests or Mann–Whitney *U* tests when appropriate. Categorical variables were statistically compared by chi‐square test or Fisher's exact test. *P* values <0.05 were considered statistically significant. The data were analysed by IBM SPSS software (IBM Corp. Released 2019. IBM SPSS Statistics for Windows, Version 27.0. Armonk, NY: IBM Corp) by a cardiologist familiar with FD and a statistician.

### Ethics approval

The protocol of the present study was approved by the Research Ethics Committee of the Northern Savo Hospital District and was performed in accordance with the Declaration of Helsinki.

## Results

### Study subjects and clinical characteristics

Until 2018, 109 patients were diagnosed with FD in Finland, resulting in a prevalence of approximately 1:50000. Among the 97 Finnish patients with FD included in this study, 32 (33%) were males and 65 (67%) were females. The clinical characteristics and symptoms are presented in *Table* 1.

**Table 1:** Clinical manifestations of Fabry disease in hemizygous males and heterozygous females

	Hemizygous males (*n* = 32)	Heterozygous females (*n* = 65)	*P*‐value males vs. females
Age at Fabry disease diagnosis, years	29 (17; 4–78)	41 (20; 3–80)	0.185
Age at the end of the follow‐up, years	42 (16; 10–86)	53 (19; 10–82)	0.334
<18 years	3 (9%)	3 (5%)	
<30 years	7 (22%)	12 (18%)	
≥30 years	25 (78%)	53 (82%)	
Phenotype			
Classic	21 (66%)	n/a	
Late‐onset	8 (25%)	n/a	
Intermediate (p.T410A)	3 (9%)	n/a	
Asymptomatic	0 (0%)	3 (5%)	
Acroparesthesia	15 (47%)	31 (48%)	0.598
Cornea verticillata	12/22 (55%)	30/50 (60%)	0.665
Angiokeratoma	23 (72%)	46 (71%)	0.683
Gi‐symptoms			
Abdominal pain	21 (66%)	32 (49%)	0.169
Diarrhoea	15 (47%)	25 (38%)	0.252
Kidney transplant	3 (9%)	0 (0%)	0.012
Renal biomarkers			
eGFR, mL/min/1.73 m^2^	93 (27)	85 (24)	0.522
Creatinine, μmol/L	87 (30)	71 (26)	0.213
Albuminuria: mild (25–300 mg/L)	9 (28%)	17 (26%)	
Severe (>3 g/L)	4 (13%)	5 (8%)	
Medication			
ERT	28 (88%)	37 (57%)	0.08
Migalastat	3 (9%)	5 (8%)	0.760

The data are the mean (SD; range) or number (percentages). The mean age of Finnish Fabry patients was 49 years (SD 19; 10–86 years) at the end of the follow up on 17 October 2018. The mean follow‐up period was 12 years. eGFR normal ranges: 18–39 y: >89 mL/min/1.73 m^2^, 40–49 y: >83 mL/min/1.73m^2^, 50–59 y: >77 mL/min/1.73 m^2^, 60–69 y: >69 mL/min/1.73 m^2^, ≥ 70 y: >59 mL/min/1.73 m^2^; Creatinine, normal range in males 60–100 μmol/L and in females 50–90 μmol/L; ERT, enzyme replacement therapy; *n*, number; normal ranges: 18–39 years: >89 mL/min/1.73 m^2^; SD, standard deviation.

Normal estimated glomerular filtration rate, normal ranges: ≥70 years: >59 mL/min/1.73 m^2^; 40–49 years: >83 mL/min/1.73 m^2^; 50–59 years: >77 mL/min/1.73 m^2^; 60–69 years: >69 mL/min/1.73 m^2^; Creatinine, normal range in males 60–100 μmol/L and in females 50–90 μmol/L; eGFR, estimated glomerular filtration rate; ERT, enzyme replacement therapy; *n*, number; normal ranges: 18–39 years: >89 mL/min/1.73 m^2^; SD, standard deviation.

The patients were diagnosed at a mean age of 37 years (range 3–80). Males were diagnosed significantly earlier than females, with mean ages at diagnosis being 29 and 41 years (*P* < 0.001), respectively. The mean age at the end of this follow‐up study was 49 years (SD 19; 10–86, 42 years for males and 52 years for females), and the mean follow‐up time was 12 years. Six individuals were aged less than 18 years (3 males aged 10–17 years, 3 females aged 10–15 years). Symptoms and findings of classic FD were common.

### Nephropathy

The creatinine levels were normal in most patients. Severe proteinuria was diagnosed in 13% of FD males and 8% of FD females (*Table* 1). Three (9%) males but none of the females developed end‐stage kidney disease. Three males received a kidney transplant, one with the *GLA* deletion encompassing exons 4–7 in his 20s, another with the p.R227Q variant in his 40s, and one with the p.P409A variant in his 40s.

### Medical treatment

ERT was started for 88% of FD males at the mean age of 35 years, and for 57% of FD females at the mean age of 52 years. Migalastat was used in few patients only.

### Lyso‐Gb_3_

See Data S1.

### Genetics

In total, 22 different *GLA* variants were identified in the 26 families with FD (*Table* *2*). Five (23%) of these variants were nonsense variants, and 13 (57%) were missense variants. Initially, 20 of 22 variants were classified as disease‐causing, and reassessment led to the downgrading of p.L311F as a VUS. We previously reported that the p.A143T variant with controversial interpretation^21^ was the cause of late‐onset FC in the Finnish family included in this study.^20^

**Table 2:** All *GLA* variants detected in the 97 affected individuals with FD from 26 Finnish families

Variant no.	M/F, *n*	Amino acid change		Nucleotide change	Variant type	Pathogenicity	Phenotype
1	4/10	R227X^a^	p.Arg227Ter	c.679C > T	Nonsense	P	Classic
2	6/7	**A143T** ^b^	p.Ala143Thr	c.427G > A	Missense	VUS/LP^c^	Late‐onset
3	2/7	**P409A**	p.Pro409Ala	c.1225C > G	Missense	P	Classic
4	3/5	Q283X	p.Gln283Ter	c.847C > T	Nonsense	P	Classic
5	2/5	T410I^a^	p.Thr410Ile	c1229C > T	Missense	P	Classic
6	3/3	T410A	p.Thr410Ala	c.1228A > G	Missense	P	Intermediate^d^
7	2/3	A15E	p.Ala15Glu	c.44C > A	Missense	P	Classic
8	1/4	R220X	p.Arg220Ter	c.658C > T	Nonsense	P	Classic
9	2/2	H46R	p.His46Arg	c.137A > G	Missense	LP	Classic
10	0/4	L311F	p.Leu311Phe	c.931C > T	Missense	VUS	n/a
11	1/3	Deletion encompassing exons 4–7			Deletion	P	Classic
12	1/1	W204X	p.Trp204Ter	c.611G > A	Nonsense	P	Classic
13	1/2	R227Q	p.Arg227Gln	c.680G > A	Missense	P	Classic
14	1/2	W349X	p.Trp349Ter	c.1046G > A	Nonsense	P	Classic
15	1/1	C94Y	p.Cys94Tyr	c.281G > A	Missense	LP	Classic
16	1/1	L275fs	p.Leu275fs	c.823delC	Frameshift	LP	Classic
17	0/1	H46Y	p.His46Tyr	c.136C > T	Missense	P	n/a
18	0/1	c.764_766delATG			Deletion	P	n/a
19	0/1	E341G	p.Glu341Gly	c.1022A > G	Missense	LP	Classic^e^
20	0/1	G43S	p.Gly43Ser	c.127G > A	Missense	LP	n/a
21	0/1	T282P	p.Thr282Pro	c.844A > C	Missense	LP	n/a
22	1/0	NA		c.640–814 T > C	Intronic	VUS	Late‐onset

Variants were classified according to ACMG. Phenotype was clinically defined based on the phenotype of affected Finnish males, their characteristic symptoms, long‐term disease manifestations and enzyme activity. Phenotype is stated as n/a, not definable, if only females are affected.

F, female; LP, likely pathogenic; M, male; NA, not available; P, pathogenic; VUS, variant of uncertain significance.

^a^ Variant in two families.

^b^ Variant in three families.

^c^ Variant caused late‐onset Fabry cardiomyopathy in a Finnish family, see Valtola K, *et al*. *Heart* 2020;**0**:1–7. doi:10.1136/heartjnl‐2019‐315933.

^d^ Variant caused intermediate disease in a Finnish family, see Valtola K, *et al*. *Open Heart* 2023**;10**:e002251. doi:10.1136/openhrt‐2023‐002251.

^e^ Defined as classic because subject's brother not included in this study cohort has classic FD.

The three most common variants (p.R227X, p.A143T and p.P409A) were identified in more than one family, p.143T in three families and the other two in two families. All other variants were found in single families.

### Fabry disease phenotypes

With a comprehensive assessment of FD phenotypes in males (*n* = 32, *Tables* *1* and *2*), we classified 19 (59%) with the classic FD phenotype, 10 (31%) with the late‐onset phenotype, and three (9%) males with *GLA* p.T410A as the intermediate FD phenotype.^9^ Among the 65 female patients, all but three (two teenage girls and one female in her 70s, all with p.A143T) were diagnosed with symptomatic FD.^20^

### CMR findings and cardiac biomarkers in FC

FC was diagnosed in approximately half of all patients (21 (66%) males and 32 (49%) females), manifesting on average over 10 years earlier in males (mean age at 41 years, vs. females 55 years, *P* < 0.001). Cardiomyopathy was diagnosed in males since their twenties and in females since their 40s, earlier in subjects carrying deletions or nonsense variants (data not shown). However, in a female carrying the missense p.T410A, an increased cardiac mass and decreased T1 time indicated that FC had been present since her 30s. In females, lyso‐Gb_3_ values were nominally greater in subjects with FC than in those without FC, but the difference was not statistically significant (data not shown).

*Table* *3* shows the latest CMR findings and cardiac biomarkers in patients with FC. NT‐proBNP and TnT were highly variable from normal to very high values. On CMR, most FC patients presented moderate to severe left ventricular hypertrophy (mean maximal LV wall thickness 15 mm, range up to 26 mm) with preserved left ventricular ejection fraction, normal LV dimensions and LVMI. Late gadolinium enhancement was detected in most patients with FC and was particularly prevalent (85%) and severe in females.

**Table 3:** Latest CMR findings and cardiac biomarkers in patients with cardiomyopathy (*n* = 53) by sex

	Hemizygous males (*n* = 21)	Heterozygous females (*n* = 32)	*P*‐value males vs. females
Age at Fabry disease diagnosis, years	35 (15)	52 (17)	<0.001
Age at cardiomyopathy diagnosis, years	41 (13)	55 (9)	<0.001
Cardiac biomarkers			
NT‐proBNP, ng/L	99 (50–1250)	452 (13–10 357)	0.020
TnT, ng/L	16 (0.3–64)	34 (9–442)	0.153
Age at latest CMR,^a^ years	41 (10)	61 (9)	<0.001
LVEDVI, mL/m^2^	80 (25)	75 (28)	0.633
LVESVI, mL/m^2^	24 (10)	25 (15)	0.834
LVMI, g/m^2^	81 (20)	72 (15)	0.326
max LVWT, mm	16 (4)	15 (3)	0.575
LVEF, %	66 (11)	68 (10)	0.518
LGE,^b^ *n* (%)	8/14 (57)	17/20 (85)	0.191
LGE, *n* (%)			
No	6 (43)	3 (15)	
Mild	5 (36)	10 (50)	
Severe	3 (21)	7 (35)	

The data are the mean (SD;range) or number (percentages).

LGE, late gadolinium enhancement; LVEDVI, left ventricular end diastolic volume indexed to body surface area (normal values for males 57–105 mL/m^2^ and for females 56–96 mL/m^2^); LVEF, left ventricular ejection fraction, normal EF ≥ 50%; LVESVI, left ventricular end systolic volume indexed to body surface area (normal values for males 14–38 mL/m^2^ and for females 14–34 mL/m^2^); LVMI, left ventricular mass index (normal values for males 49–85 g/m^2^ and for females 41–81 g/m^2^); max LVWT, maximal left ventricular wall thickness in end‐diastole (normal <12 mm); NT‐proBNP, NT‐pro brain natriuretic peptide (normal range < 50 years: 0–450 ng/L, 50–70 years: 0–900 ng/L, over 75 years: 0–1800 ng/L; SD, standard deviation; TnT, troponin T (normal range 0–15 ng).

^a^ Of patients with Fabry cardiomyopathy 17 of 21 males (81%) and 26 of 31 females (84%) underwent CMR at least once.

^b^ LGE was available in 14 of 17 CMR in males and 20 of 26 CMR of females.

### Cardiovascular manifestations

**Figure 2:**
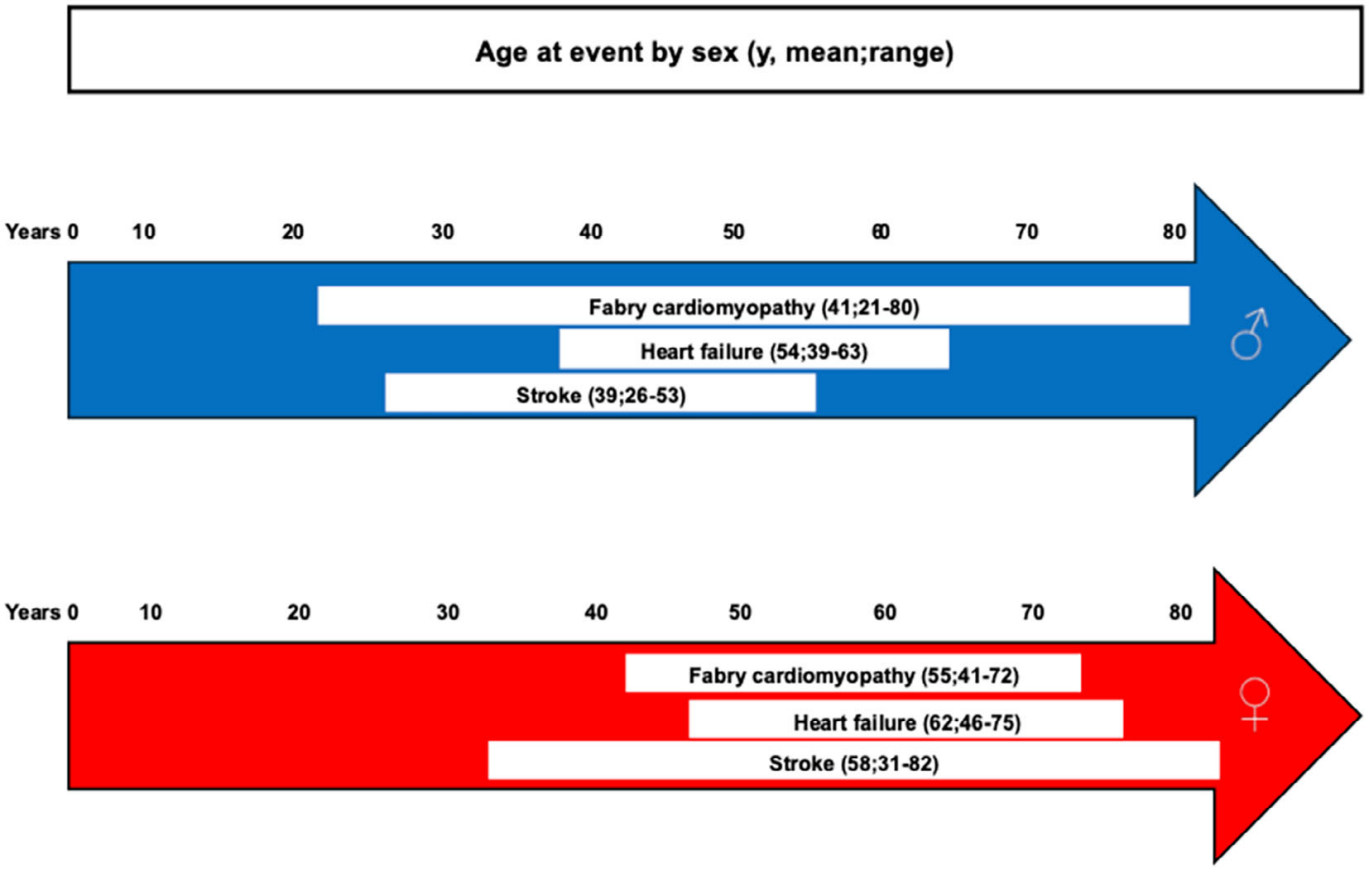
Age of Finnish Fabry patients at the onset of major cardiovascular events by sex.

The cardiovascular manifestations of all FD patients are shown in *Table* *4* and *Figure* *2*. In contrast to cardiomyopathy, the prevalence of hypertension, coronary artery disease, and the need for a pacemaker or ICD was low. Atrial fibrillation (AF), HF and stroke developed in 15–22% of patients. Among FC patients with symptomatic heart failure (HF), most males (67%) had HF with reduced ejection fraction (< 50%), whereas the majority of the females (83%), had HF with preserved left ejection fraction.

**Table 4:** Cardiovascular manifestations of Finnish Fabry disease patients (*n* = 97)

	Hemizygous males (*n* = 32)	Heterozygous females (*n* = 65)	*P*‐value, males vs. females
Hypertension^a^	3/28 (11%)	10/61 (16%)	0.499
Coronary artery disease^b^	3 (9%)	4 (6%)	0.550
Cardiomyopathy^c^	21 (66%)	32 (49%)	0.127
Age at diagnosis, years	41 (13)	55 (9)	
Atrial fibrillation	3 (9%)	14 (22%)	0.522
Heart failure	3 (9%)	12 (18%)	0.166
Age at diagnosis, years	54 (13)	62 (8)	0.294
LVEF ≥ 50%	1 (33%)	10 (83%)	
LVEF < 50%	2 (67%)	2 (17%)	
Stroke	7 (22%)	14 (22%)	0.970
Age at diagnosis, years	39 (9)	58 (15)	
Bradycardia pacemaker	2 (6%)	9 (14%)	0.654
ICD	1 (3%)	1 (2%)	0.202

The data are number (percentages) or the mean (SD).

CD, implantable cardioverter defibrillator; LVEF, left ventricular ejection fraction; SD, standard deviation.

^a^ The patient has hypertension if the resting blood pressure is ≥ 140/85 mmHg.

^b^ Coronary artery disease is defined as ≥50% narrowing in ≥1 epicardial vessel on coronary angiography.

^c^ Fabry cardiomyopathy is defined by maximal left ventricular wall thickness of ≥13 mm measured in end‐diastole, measured on echocardiography or CMR. In one female case, cardiomyopathy was diagnosed by elevated left ventricular mass index and decreased T1 time on CMR.

In 53 patients with cardiomyopathy, cardiovascular outcomes were particularly frequent; 16 (30%) developed AF, 17 (32%) stroke and 14 (26%) HF during the follow‐up. Only patients with FC required a bradycardia pacemaker or an ICD or developed end‐stage renal disease. In contrast, in 44 FD patients without FC, AF and HF were diagnosed in only one female, and one male and three females had a stroke.

### Natural history and mortality

During the follow‐up, nine patients (9%) died. The sex, age at death, FD causing variant, causes of death and natural history of FD are shown in *Table* *5*. Eight of the nine deceased had been diagnosed with FC. Three died from HF, and three died from stroke. The typical natural history consisted of a sequence of diagnoses of cardiomyopathy, AF, HF, and stroke, in variable combinations, ultimately leading to death at a mean age of 48 years in males and 75 years in females, most often within 10 to 15 years of FC diagnosis.

**Table 5:** Natural history of the nine Finnish Fabry patients who died during the follow‐up

Gender/age at death, years GLA variant		Age at diagnosis, years	Age when ERT started, years	Reported cause of death	Medical history	
1	M/40 p.Q283X	24	39	Stroke	39 years	Fabry cardiomyopathy with heart failure
2	M/47 p.A15E	21	31	GI‐bleeding	37 years	Fabry cardiomyopathy
3	M/57 p.P409A	40	‐	Lymphoma	45 years 46 years 48 years 49 years	3rd degree AV block and heart failure, pacemaker with ICD, stroke Severe kidney failure, dialysis Renal transplantation Fabry cardiomyopathy
4	F/68 p.Q283X	48	63	Heart failure	54 years 58 years 67 years	Fabry cardiomyopathy Coronary artery disease Heart failure
5	F/69 p.G43S	48	62	Heart failure	54 years 61 years 65 years 69 years	Fabry cardiomyopathy Heart failure Myocardial infarction and VF AF and bradycardia pacemaker TIA
6	F/74 p.T410A	67	69	Stroke	61 years 67 years 74 years	Fabry cardiomyopathy Heart failure, AF SSS, bradycardia pacemaker Stroke
7	F/74 p.L311F^a^	50	61	Multiple myeloma	49 years 56 years 57 years 62 years 67 years 70 years	Fabry cardiomyopathy Stroke TIA NSVT AF and SVT TIA, severe proteinuria, and multiple myeloma diagnosed
8	F/82 Deletion of exons 4–7	76	‐	Heart failure	57 years 63 years 75 years	AF and SVT, first stroke Fabry cardiomyopathy VF and heart failure, pacemaker with ICD due to VT, second stroke
9	F/82 p.R227X	80	‐	Stroke	77 years 82 years	AF Stroke

AF, atrial fibrillation; AV, atrioventricular; ERT, enzyme replacement therapy; GI‐bleeding, gastrointestinal bleeding; ICD, implantable cardioverter defibrillator; NSVT, non‐sustained ventricular tachycardia; SSS, sick sinus syndrome; SVT, supraventricular tachycardia; TIA, transient ischaemic attack; VF, ventricular fibrillation; VT, ventricular tachycardia.

^a^ VUS, variant of unknown significance.

## Discussion

### Principal findings

On the basis of the present study, the prevalence of FD in Finland is approximately 1:50 000 individuals. Most disease‐causing *GLA* variants were found in single families, and the predominant phenotype was classic. Almost all female patients were symptomatic. Cardiomyopathy with subsequent AF, HF and stroke was common in both sexes, manifesting since their twenties in males and since their 40s in females. LGE, indicating permanent cardiac damage,^6^, ^22^ was particularly common and severe in females. During the 12‐year follow‐up, nine patients died, all but one with FC. FC contributed to premature death in most patients. FD significantly shortened life expectancy in both sexes, as male patients died in their 40s and females in their 70s.

### In the context of the current literature

The global estimated prevalence of classical FD in males is 1 in 50 000 males.^23^ However, FD, especially late‐onset FD, is underdiagnosed in many countries. In newborn screenings in Italy and Taiwan, the prevalence of late‐onset FD causing *GLA* variants was 1:3000 and 1:1200 males, respectively.^10^ Recent UK Biobank research revealed a prevalence of pathogenic *GLA* variants associated with late‐onset FD of 1:5732.^24^ The prevalence of FD in Finland according to the present study is approximately 1:50 000. Despite of the unique inheritance in Finland, founder variants were not detected. Most disease‐causing *GLA* variants were found in single families. All three variants identified in more than one family have often been reported in other FD cohorts. In contrast to previous studies,^25^ classic phenotype of the disease was more common than late‐onset phenotype, which, along with the rather low observed prevalence of FD, suggests that late‐onset FD is also underdiagnosed in Finland. Late‐onset FD is challenging to diagnose, as FC, which is often the only manifestation, does not appear until early middle‐age.^10^

In the present study, almost all females carrying pathogenic/likely pathogenic *GLA* variants were symptomatic, which contrasts with the findings of historical studies suggesting that a majority of females with disease‐causing variants do not have signs or symptoms of FD.^5^ Males with classic FD are usually diagnosed with early nephropathy and strokes, followed by FC by the age of 40.^7^, ^26^ Previous studies have reported FC in 53% of males and in ≥33% of females after 30 years of age,^3^ with no significant difference in the manifestation of FC between classic and late‐onset patients.^3^, ^10^, ^11^ The present study revealed that the likelihood of developing end‐stage renal disease was low in the Finnish FD cohort, but cardiomyopathy and related cardiovascular complications were common in both sexes in both the classic and late‐onset forms of FD.^27^

There are only a few previous studies on the imaging of FC. According to a recent review, LVH is present in 43% of males and 26% of females with FD, with a mean age of onset of 39 ± 10 vs. 50 ± 11 years, and the prevalence of LVH increases with age. Intramyocardial LGE is reported in 50% of patients.^6^ In a previous nationwide study from Iceland, eight index patients from two large families were identified in a study of the genetic causes of HCM.^11^ Among the 41 total patients, 16 members of one family had the *GLA* variant p.D322 and classic FD, and 25 members of another family had the *GLA* variant p.I232Tcausing late‐onset FD. All males over 30 years of age had LVH, with maximal LV thickness ranging from 12 to 26 mm. The incidence of stroke was high, particularly in patients with the classic variant.^11^ In the present study, LVH was very common not only in men but also in women, diagnosed in half of the females since their 40s. LGE was found in most patients with FC and was more common and severe in females. The higher than historical prevalence of FC and LGE in women in the present study is probably due to the routine use of CMR in Finnish Fabry patients.

The overall frequencies of HF, AF and the need for a pacemaker corresponded to the findings of previous studies.^6^ However, in contrast to the low event rates in patients without FC, approximately one‐third of patients with FC, developed AF and stroke, one‐ fourth developed HF, and 15% died during follow‐up, emphasizing the malignant natural course of FC.^3^, ^10^, ^13^, ^28^ The typical histories of patients with FC suggest that embolic stroke, due to AF and HF caused by FC, is an important mechanism for late‐onset stroke in patients with FD.

### Clinical implications

The present study demonstrated how national collaboration allows for comprehensive follow‐up and outcome data of FD in Finland. The routine use of cardiomyopathy gene panels, including the *GLA* gene, lyso‐Gb_3_ measurements, and CMR imaging techniques, has yielded a detailed view of the natural course of FD. Our findings highlight the need for even more diligent monitoring of cardiac manifestations by regular cardiac imaging by CMR, not only in men but also in women from their 30s to their 40s. Cardiologists play an essential role in initiating specific therapies early enough to improve the prognosis of FC.^26^ Finally, the international research community is challenged by the need to find novel diagnostic biomarkers for FC and more accurate guidelines on how to monitor FD patients and when to start therapy.

### Strengths and limitations of the study

This study represents a nationwide cohort of most FD patients diagnosed in Finland by 2018, with a long mean follow‐up period. The diligently recorded patient data from medical records and the Fabry Registry allowed us to comprehensively examine the natural history and outcome of FC, as genetic, cardiac imaging, and mortality data, which are very reliable in Finland, were available.

One limitation of this study is that ERT was available in clinical practice in Finland not earlier than 2004, and it does not reflect the quality of current diagnostics and treatment of FD in Finland. Today, the availability of CMR and lyso‐Gb_3_ measurements is very high, and they are routinely used in the diagnosis and follow‐up of patients with FD.

## Conclusions

In Finnish Fabry patients, the genetic background is diverse. Cardiomyopathy is very common not only in men but also in women and is associated with AF, HF, stroke and premature death, emphasizing the malignant natural course of FC.

## Conflict of Interest

- Research grants for studying hypertrophic cardiomyopathy and Fabry disease (Academy of Finland, Finnish Foundation for Heart Research, Kuopio University Hospital, Shire, Sanofi and BMS).
- Invited lecturer in meetings of the Finnish Cardiac Society, Society for Internist in Finland, European Society of Cardiology, Scandinavian Fabry Meeting and drug companies.
- Sponsored delegate in conferences in cardiology and metabolic diseases.
- Temporary advisory board member of Sanofi, Amgen, Pfizer, Chiesi and BMS.

## Supporting information


**Data S1.** Supplementary Material.
