# Supplementary material for: Genetics, cardiac phenotype and cardiovascular outcomes in Fabry disease patients in Finland
Source: ESC Heart Fail. 2025 Jul 21;12(5):3502–11. doi: 10.1002/ehf2.15387 (PMC12450783; doi:10.1002/ehf2.15387)
Supplement: Supplementary file 1 — Data S1. Supplementary Material. [file EHF2-12-3502-s001.docx]

**SUPPLEMENTAL MATERIAL**

**The Fabry Registry**

The Fabry Registry (1) (ClinicalTrials.gov Identifier NCT00196742) is a multicenter, international, longitudinal, patient-centered, observational study open to all patients with a confirmed diagnosis of FD, irrespective of treatment status or therapy administered. It is designed to collect consistent retrospective and prospective patient data (clinical, demographic, genetic, biochemical, imaging, and therapeutic **data**) with the aim **of** **examining** the natural history of FD and the effects of ERT. **The** participation of patients and investigators is voluntary. The operations and management of the Fabry Registry are supported by an experienced and specialized team from Sanofi. It has expanded globally with **increasing** numbers of participating patients and contributing healthcare professionals comprising more than 8000 individuals with FD from all over the world.

**References**

1. Wanner C, Ortiz A, Wilcox WR, Hopkin RJ, Johnson J, Ponce E, et al. Global reach of over 20 years of experience in the patient-centered Fabry Registry: Advancement of Fabry disease expertise and dissemination of real-world evidence to the Fabry community. Mol Genet Metab. 2023;139(3):107603.

**Fabry Disease Centre of Excellence in Finland**

The Fabry Disease Centre of Excellence team visits these hospitals annually and meets the local healthcare experts responsible for Fabry patients´ care. The annual monitoring data and data from medical records are collected and recorded, with the patient's consent, in the Fabry Registry database by the Fabry Disease Centre of Excellence. Other physicians treating FD have access to the registry with special permission.

All Finnish patients enrolled in the Fabry Registry had previously given written informed consent to be included in the Fabry Registry, and to review their medical records. They are investigated and closely monitored annually according to the Finnish FD protocol in **the** clinical setting **at** Finnish University Hospitals (Helsinki, Kuopio, Turku, Tampere) or in Central Hospitals (Vaasa, Pori, Seinäjoki, Kajaani, Lappeenranta or Jyväskylä) by FD specialized cardiologists, endocrinologists, internists, nephrologists or pediatricians. If appropriate, a neurologist, nephrologist, ophthalmologist, psychiatrist, pulmonologist, and dermatologist are consulted.

**Finnish national Fabry disease protocol**

The Finnish Fabry disease protocol (1, 2) includes careful patient examination by **a** specialist (cardiologist, internist, nephrologist, neurologist or pediatrician), an interview (symptom and depression questionnaire, quality of life measurements, and cognitive tests) and several laboratory tests (complete blood count, plasma levels of sodium, potassium, creatinine, urea, cystatin C, alanine aminotransferase, gamma-glutamyl transpeptidase, NT-pro Brain Natriuretic Peptide (NT-proBNP), troponin T, high-sensitivity C-reactive protein (hsCRP) and thyroid stimulating hormone, fasting plasma glucose, glycohemoglobin (Hba1C) and lipids, albumin-to-creatinine ratio, estimated GFR, creatinine clearance, ECG, plasma and urine levels of globotriaosylsphingosine (lyso-Gb_3_) and antibodies if on ERT) at least annually. In addition, individually, at the discretion of a physician, **a** cardiac stress test, 24-hour ECG monitoring, cardiac ultrasound, **and** cardiac MRI including LGE and, in some centers, also T1 mapping, brain MRI, spirometry, audiogram and an examination by an ophthalmologist and dermatologist will be performed.

**References**

1. Kantola I, Penttinen M, Nuutila P, Viikari J. Fabryn tauti. *Duodecim* 2012;128:729-39.
2. Kantola I, Walls S. Fabryn tauti ja sen hoito. *Sic!:4 (Lääkealan turvallisuus- ja kehittämiskeskus Fimea,* 12 / 2016)

**Fabry disease phenotype classification**

In this study, **the** phenotype of FD was defined in male patients, **on the basis of** characteristic symptoms, long-term disease manifestations and enzyme activity, and **the** type of genetic defect as suggested by European FD experts. (7, 17, 18). **The** phenotype was considered classic if the patient had a known classical *GLA* variant, α-Gal A activity < 5% and/or symptoms such as severe intermittent limb pain (acroparesthesia), hypohidrosis or anhidrosis, **and** gastrointestinal (GI) symptoms were present already in childhood or adolescence and/or a patient was detected with cornea verticillata and/or angiokeratomas.(4) **The** phenotype was defined as late-onset when an adult male patient had symptoms or clinical findings of FD, a known pathogenic or likely pathogenic variant, α-Gal A activity ≥ 5%, and no signs or symptoms of FD before the age of 18 years.(1) **The** phenotype was defined as intermediate when there were both classic and late-onset males in the same family without other *GLA* mutations explaining the differences.(5) Phenotype was not definable if only females were affected. Females were classified as either symptomatic or asymptomatic.

**References**

1. Arends M, Wanner C, Hughes D, Mehta A, Oder D, Watkinson OT, et al. Characterization of Classical and Nonclassical Fabry Disease: A Multicenter Study. J Am Soc Nephrol. 2017;28(5):1631-41.
2. Germain DP. Fabry disease. Orphanet J Rare Dis. 2010;5:30.
3. Germain DP, Altarescu G, Barriales-Villa R, Mignani R, Pawlaczyk K, Pieruzzi F, et al. An expert consensus on practical clinical recommendations and guidance for patients with classic Fabry disease. Mol Genet Metab. 2022;137(1-2):49-61.
4. Arbelo E, Protonotarios A, Gimeno JR, Arbustini E, Barriales-Villa R, Basso C, et al. 2023 ESC Guidelines for the management of cardiomyopathies. Eur Heart J. 2023;44(37):3503-626.
5. Valtola K, Hedman M, Kantola I, Walls S, Helisalmi S, Maria M, et al. Late-onset and classic phenotypes of Fabry disease in males with the GLA-Thr410Ala mutation. Open Heart. 2023;10(1).

**Alpha-galactosidase A enzyme activity and lyso-Gb_3_ level**

**The** measurement of plasma/leukocyte α-galactosidase A enzyme (α-Gal A) activity was performed by the individual centers at least once for almost all the research subjects in the diagnosis phase prior to any treatment. The methods for measuring enzyme activity and the cut-off values for **the** definition of diagnosis were defined by each diagnostic laboratory. Measurements of plasma lyso-Gb_3_ before FD-specific treatment **have been** available since 2014 for 53% of the study patients. Plasma lyso-Gb_3_ values during ERT or migalastat treatment were available **for** 57% of the patients.

**Cardiac imaging**

The imaging studies were performed as a part of normal Fabry outpatient follow-up in hospitals.^20^ Cardiac ultrasound imaging was performed at least once on 30 of 32 males (94%) and on 59 of 65 females (91%). Cardiac magnetic resonance (CMR) imaging was performed at least once in 25 of 32 males (78%) and 48 of 65 females (74%). Among patients diagnosed with FC, 17 of 21 males (81%) and 27 of 32 females (84%) underwent CMR at least once. CMR-derived late gadolinium enhancement (LGE) was investigated in 14 males and 20 females with FC. CMRs were performed using a minimum 1.5 T whole-body scanner. Cardiologists or imaging cardiologists and cardiac radiologists performed and interpreted cardiac imaging findings according to current guidelines.

**Lyso-Gb_3_**

The mean plasma lyso-Gb_3_ level before FD treatment was 55 ng/l (SD 44; range 2-110; normal 0-3 ng/l) in males (data available for 34% of them) and 8 ng/l (SD 5; range 1-19; normal 0-3ng/l) in females (available for 62% of them). During ERT or migalastat treatment, the mean plasma lyso-Gb_3_ was 23 ng/l (SD 21; range 2-71ng/l) in males and 8 ng/l (SD 5; range 3-25 ng/l) in females.
